# Stressors in university life and anxiety symptoms among international students: a sequential mediation model

**DOI:** 10.1186/s12888-023-05046-7

**Published:** 2023-08-01

**Authors:** Yue Wang, Xiaobin Wang, Xuehang Wang, Xiaoxi Guo, Lulu Yuan, Yuqin Gao, Bochen Pan

**Affiliations:** 1 Center for Reproductive Medicine, Department of Obstetrics and Gynecology, Shengjing Hospital of China Medical University, No. 39 Huaxiang Road, Tiexi District, Shenyang, 110022 China; 2 The Fourth Affiliated Hospital of China Medical University, Shenyang, China; 3 International Education School, China Medical University, Shenyang, China; 4 School and Hospital of Stomatology, China Medical University, Liaoning Provincial Key Laboratory of Oral Diseases, Shenyang, China

**Keywords:** Anxiety, Perceived stress, Self-efficacy, Students, University

## Abstract

**Background:**

Anxiety is a common mental health problem among university students, and identification of its risk or associated factors and revelation of the underlying mechanism will be useful for making proper intervention strategies. The aim of our study is to test the sequential mediation of self-efficacy and perceived stress in the association between stressors in university life and anxiety symptoms.

**Methods:**

A cross-sectional study design was adopted and a sample of 512 international students from a medical university of China completed the survey with measurements of stressors in university life, self-efficacy, perceived stress and anxiety symptoms.

**Results:**

We found that 28.71% of the international students had anxiety symptoms, and stressors in university life were positively associated with anxiety symptoms (*β* = 0.23, *t* = 5.83, *p* < 0.01). Moreover, sequential mediating role of self-efficacy and perceived stress in the association between the stressors and anxiety symptoms was revealed.

**Conclusions:**

Our study provided a new perspective on how to maintain the mental health, which suggested that self-efficacy improvement and stress reduction strategies should be incorporated in the training programs to support students.

## Background

There has been an increasing interest in mental health of university students, and one of the growing concerns is the high prevalence of stress and anxiety in this population [1–4]. According to the WHO survey project about mental health problems in university students, generalized anxiety was highly prevalent in students across countries [5]. As anxiety can seriously affect students’ social function, academic achievement or even physical health, efforts should be made to identify its risk factors and illuminate the mechanism of interactions so that proper intervention strategies may be made. International students are a special population in universities, because they may have to deal with the culture differences and encounter more difficulties. Thus, their mental health is of great concern. However, studies focusing on the mental health of international students in China have been limited.

### Relationship between stressors and anxiety symptoms

Studies suggest that stress is one of the risk factors for anxiety [6]. Higher levels of stress among university students have been found associated with mental health problems [7–9]. There are many sources of stress that university students may experience, and the students must learn to balance the competing demands. Demands on an individual made by the external or internal environmental stimuli that affect the balance are defined as stressors [10]. In recent years, more and more researchers begin to focus on the stressors that university students are facing and the coping strategies students adopt. Stressors encountered by university students are categorized differently in different studies, but some major sources have been well recognized, such as the health issues, environmental problems, academic difficulties, financial pressure and interpersonal relation problems [11, 12]. Among these sources, academic difficulties were viewed as the primary sources of stress and were shown contributing to a variety of mental health problems in many researches [13, 14]. For international students, however, the stressors might be different, because they may face issues different from those of their domestic peers. Therefore, studies specific on the stressors perceived by international students are needed. In this study, based on the above observations, our first hypothesis (H1) is: Stressors in university life are positively and significantly associated with anxiety symptoms among international students.

However, researches have shown that the same stressors to one individual may not be stressful to another. Other factors may also contribute to the process. Lazarus and colleagues described this phenomenon in their transactional model of stress, and they pointed out that cognitive appraisals play an important role in the process to determine the presence or the severity of a stressor [15]. In this model, stress is defined as a transaction between an individual and the environment, and is generated by subjective cognitive judgement of the potential impact of a stressor on future functioning [16]. The process begins when a stressor represents a threat (primary appraisal) that activates a cognitive process for the individual to assess the degree of harm or loss, and then leads to a secondary appraisal in which the individual evaluates his or her resources to cope with the stressor. A stress response is elicited when the perceived demands outweigh the perceived resources. Therefore, it is the character of the individual rather than the environment that makes a difference in the meaning of a stressor. In addition, stress outcomes were known to involve physiological, emotional, behavioral and cognitive reactions [15].

### Relationships among stressors, self-efficacy and anxiety symptoms

Self-efficacy is grounded in the social cognitive theory which emphasizes that the individual regulates his or her motivation and behavior through self-assessment [17]. General self-efficacy is defined as the degree to which individuals believe they are capable of dealing with challenging situations and is the mechanism through which individuals apply their existing knowledge and experience [18]. If individuals have a strong sense of self-efficacy, they will trust their ability to actively control stressors in the environment, which will motivate them to take action [19]. This is very similar to the appraisal concept in the transactional model of stress [19]. Studies revealed that the lower level of self-efficacy was related to mental disorders [20, 21]. This may be true for the university students as students with more anxiety symptoms showed lower level of self-efficacy [22, 23]. Individuals with higher levels of self-efficacy tend to experience more positive emotions, whereas those with lower levels of self-efficacy are more likely to experience more anxiety [24]. A possible explanation is that people who have lower perception of self-efficacy to control life and thoughts cannot help but be anxious at the thought of how to deal with the stressors [19]. Therefore, self-efficacy appears to be an effective protective factor against the negative psychological effects such as anxiety induced by stressors, and thus we propose the following hypotheses: (H2) Stressors in university life are negatively and significantly associated with self-efficacy among international students; (H3) Self-efficacy is negatively and significantly associated with anxiety symptoms among international students; (H4) Stressors in university life have a significant indirect effect on anxiety symptoms via self-efficacy among international students.

### Relationships among stressors, perceived stress and anxiety symptoms

Appraisal or perception of stress is another factor that may mediate the association between the stressors and psychological responses. Based on the transactional model of stress, stress represents an imbalance between abilities of individuals and demands of environment, and the results of the transaction could lead to negative psychological outcomes [15]. Therefore, the effect of stressors depends on the perception of stress [16]. Some study results confirmed the presence of such a mechanism. For example, McCuaig Edge investigated the impact of combat exposure on psychological distress of military personnel and found the mediation effect of cognitive appraisal in the association [25]. Besharat et al. conducted a survey regarding anxiety among Iranian university students, and found that perceived stress played a mediating role in the association between facing existential issues and anxiety [26]. Zhang et al. examined the relationships of sleep quality and anxiety/depression among nursing students of a public university in the United States, and found that perceived stress not only mediated the association between sleep quality and anxiety symptoms, but also the association between sleep quality and depression symptoms [27]. These results strongly suggest that the appraisal or perception of stress can be the factor that determines whether the stressors will result in psychological responses or not. As a result, we posit the following hypotheses: (H5) Stressors in university life are positively and significantly associated with perceived stress among international students; (H6) Perceived stress is positively and significantly associated with anxiety symptoms among international students; (H7) Stressors in university life have a significant indirect effect on anxiety symptoms via perceived stress among international students.

### Relationship between self-efficacy and perceived stress

A review of the literature related to self-efficacy and stress revealed a significant relationship between individuals’ self-efficacy and their effectiveness in coping with stress [24]. Self-efficacy is related to experiencing less negative emotions in risky situations and appraising the stressors as challenges rather than threats [20]. Individuals with higher level of self-efficacy believe they are capable of dealing with their demands, and this belief may result in adopting positive approaches and perceiving less stress in life. According to the transactional model of stress, self-efficacy may play a significant role in the primary and secondary appraisals which will lead to a decline in perceived stress, and then result in less negative psychological outcomes. Thus, we posit the following hypotheses: (H8) Self-efficacy is negatively and significantly associated with perceived stress among international students; (H9) Stressors in university life have a significant indirect effect on anxiety symptoms via self-efficacy and then perceived stress among international students.

**Fig. 1:**
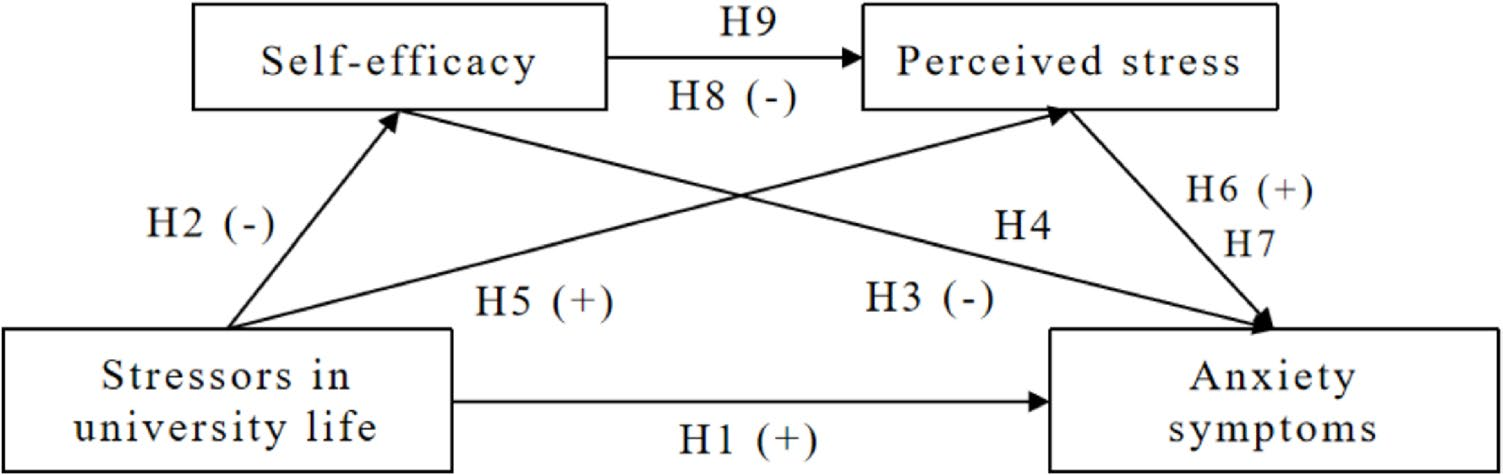
Conceptual framework of this study

Based on the above mentioned theoretical assumptions and research results, we have constructed the conceptual framework of this study (Fig. 1). We hope that this conceptual framework will become the theoretical basis for exploring intervention measures to prevent or manage mental health problem such as anxiety for the international students.

## Methods

### Study design and subjects

The present study was a cross-sectional design and a cluster sampling was adopted. Data were collected from the international students of China Medical University in November 2020. The inclusion criteria of the potential participants were (1) able to get access to internet, (2) a current student of the University, (3) able to read, fully understand and answer the survey questions. One thousand and fifteen students who met the inclusion criteria were initially contacted via electronic email. Then, in the online survey, there was a brief explanation about the study, and the participants were asked to complete an informed consent agreement, in which they were made aware that participation was completely voluntary. Research Ethics Committee of China Medical University approved our study (2020–25), and the study was performed in accordance with the Declaration of Helsinki. Finally, a total of 543 international students participated, and 512 of them were able to complete the questionnaires. The overall response rate was 50.44%.

### Measurements

#### Measurement of anxiety symptoms

The anxiety symptoms were measured with the Generalized Anxiety Disorder 7-item (GAD-7) questionnaire. The questionnaire consists of seven items for observing the frequency of anxiety symptoms with a four-point Likert scale from 0 “not at all” to 3 “almost every day” [28]. The anxiety level is reflected by the total score, where higher scores indicate more symptoms of anxiety. Scores of 5, 10 and 15 represent the cutoffs for mild, moderate and severe anxiety symptoms, respectively [29]. Previous studies have demonstrated the GAD-7 has high reliability as well as good criterion and construct validity [30–33]. The Cronbach’s alpha for this sample was 0.92.

#### Measurement of stressors in university life

Stressors in university life of international students were measured by 7 questions regarding (1) health problems, (2) financial pressure, (3) academic difficulties, (4) interpersonal relation difficulties, (5) daily life difficulties, (6) adverse life events and (7) language barrier [11, 12]. Participants answered 1 (not at all) to 4 (very serious) to the questions. The total score represents the severity of the stressors perceived by the participant. A Cronbach’s alpha of 0.80 was found for the scale in this study.

#### Measurement of self-efficacy

Self-efficacy was assessed with the General Self-efficacy Scale (GSES), which is a 10-item measure of an individual’s confidence in his or her ability to deal with stressful situations [34]. Items are scored on a four-point Likert-type scale ranges from 1 (not at all true) to 4 (exactly true), and responses are calculated to yield a total score of all item scores where higher scores indicate higher levels of self-efficacy. The GSES has good psychometric properties, and many studies have confirmed its internal consistency reliability, convergent and discriminant validity [35, 36], demonstrating that GSES is a reliable and valid measurement. The Cronbach’s alpha for GSES in the present study was 0.95.

#### Measurement of perceived stress

Perceived stress was evaluated by the 10-item version of Perceived Stress Scale (PSS-10), which is a self-report measure designed to assess the extent to which participants appraise their lives to be stressful [37]. Each item is rated on a 0 (never) to 4 (very often) Likert scale by the respondent to indicate how often the participant experienced specific feelings or thoughts. The total scores of the measure are obtained by adding the score of each item (4 items are reverse-scored) to provide a continuous measure of perceived stress, and higher scores indicate greater perceived stress. PSS-10 has demonstrated strong psychometrics. Its coefficient alpha reliability ranged between 0.84 and 0.91 in previous studies [6, 38], and in this study it was 0.87.

### Demographic characteristics

Age, gender, current place of residence (Asia/Africa/North America/Europe/Oceania) and educational background were investigated for demographic characteristics.

### Statistical analysis

SPSS 17.0 was used for the statistical analysis. Descriptive statistics including frequency distributions for the nominally scaled demographic variables provided a profile of the sample. We found the scores of GAD-7 were not normal distribution after testing the normality for continuous variables. Therefore, Mann–whitney *U* test was conducted to determine if the groups were statistically equivalent on anxiety symptoms. Spearman’s rank correlation coefficients were used to examine relationships between continuous variables.

The sequential mediation was tested using PROCESS macro program for SPSS [39], which facilitated path analysis-based mediation analyses. We verified the hypothesis model by the bias-corrected percentile bootstrap method, with 5000 resampled samples. 95% confidence intervals for the mediation effects were estimated and the results were considered significant when the 95% confidence interval did not include zero. We generated direct effect of stressors in university life on anxiety symptoms and indirect effects of stressors in university life on anxiety symptoms through the mediators (self-efficacy and perceived stress) in the mediation using the model 6 of PROCESS. There were three routes of indirect effects in the sequential mediation model. When the direct effect became non-significant but the indirect effect was significant, full mediation was established. Partial mediation was confirmed if both effects are significant [40]. Continuous variables were all centralized before the model was validated to avoid multicollinearity. Two-tailed alpha 0.05 was used for significance testing purposes.

## Results

### Descriptive statistics

The descriptive statistics of the sample are shown in Table 1. Overall, 147 (28.71%) students had anxiety symptoms, including 93 (18.16%) mild, 33 (6.45%) moderate and 21 (4.10%) severe cases.

**Table 1:** Descriptive statistics

Variables	*N* (%)
GAD-7	
No anxiety symptom	365 (71.29)
Mild anxiety symptoms	93 (18.16)
Moderate anxiety symptoms	33 (6.45)
Severe anxiety symptoms	21 (4.10)
Gender	
Male	272 (53.12)
Female	240 (46.88)
Current place of residence	
Asia	438 (85.55)
Other continents	74 (14.45)
Educational background	
Undergraduate	453 (88.48)
Master’s or Doctoral	59 (11.52)
	Mean ± *SD*
Age	22.77 ± 3.62
Stressors in university life	11.35 ± 3.19
GSES	31.08 ± 6.82
PSS-10	16.53 ± 5.95

### Severity of stressors

Stressors and their severity perceived by the international students are presented in Table 2. Financial pressure and language barrier were the most prominent stressors affecting 72.07% and 69.34% of the students, respectively.

**Table 2:** Stressors and their severity perceived by the international students

Stressors	Severity of the stressors (*N* (%))			
	Not at all	A little	Moderate	Very serious
Health problems	470 (91.80)	40 (7.81)	2 (0.39)	0 (0)
Financial pressure	143 (27.93)	188 (36.72)	132 (25.78)	49 (9.57)
Academic difficulties	328 (64.06)	135 (26.37)	37 (7.23)	12 (2.34)
Interpersonal difficulties	382 (74.61)	97 (18.94)	24 (4.69)	9 (1.76)
Daily life difficulties	292 (57.03)	157 (30.67)	49 (9.57)	14 (2.73)
Adverse life events	276 (53.91)	143 (27.93)	76 (14.84)	17 (3.32)
Language barrier	157 (30.66)	215 (41.99)	109 (21.30)	31 (6.05)

### Differences of anxiety symptoms in categorical variables

The differences of GAD-7 scores in categorical variables are shown in Table 3. There was no difference between the groups.

**Table 3:** Differences of GAD-7 scores in categorical variables

Variables	Median	Mann–whitney *U*	*p*
Gender			
Male	1	31168.50	0.36
Female	2		
Current place of residence			
Asia	2	13989	0.06
Other continents	1		
Educational background			
Undergraduate	2	11332	0.05
Master’s or Doctoral	0		

### Correlations among continuous variables

The correlations among continuous variables are shown in Table 4. Age, stressors in university life, perceived stress and self-efficacy were all significantly correlated with GAD-7 score. In addition, stressors in university life and PSS-10 negatively correlated with GSES. Finally, stressors in university life positively correlated with PSS-10, while age negatively correlated with PSS-10.

**Table 4:** Correlations among continuous variables

Variable	1	2	3	4
1. Age				
2. Stressors in university life	0.01			
3. PSS-10	-0.09^*^	0.40^**^		
4. GSES	0.04	-0.24^**^	-0.35^**^	
5. GAD-7	-0.09^*^	0.41^**^	0.56^**^	-0.23^**^

^*^p < 0.05

^**^p < 0.01

### Results of the sequential mediation model testing

**Fig. 2:**
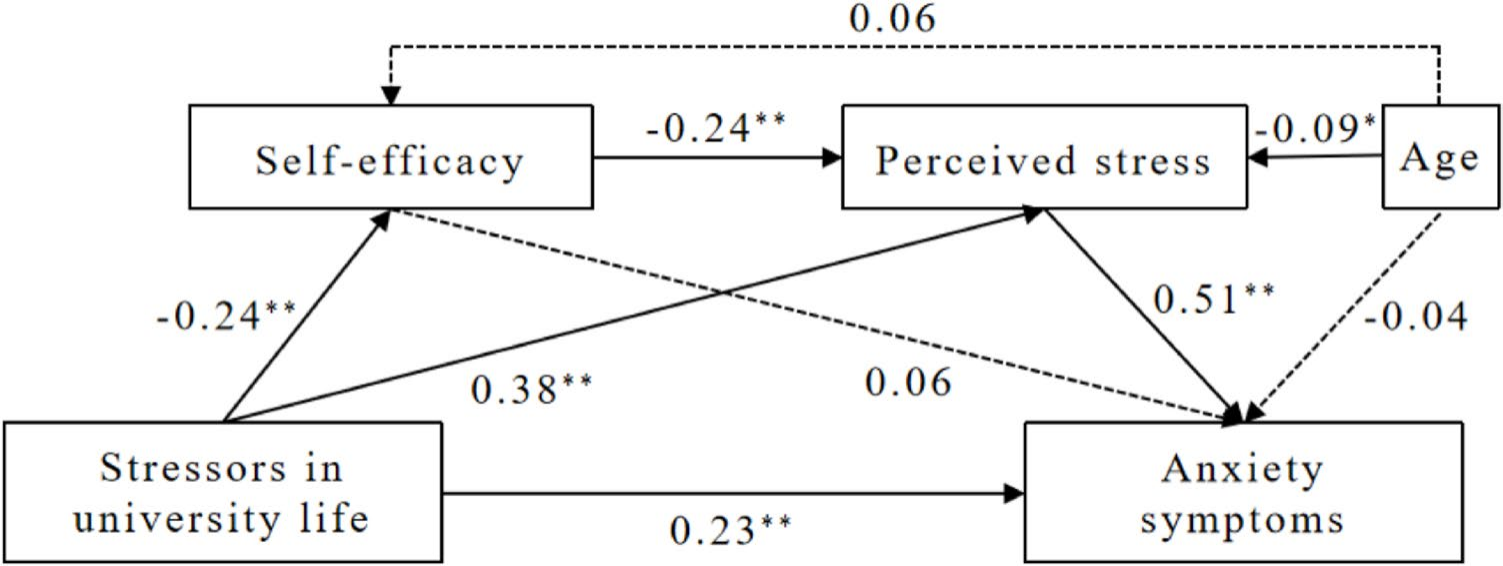
Sequential mediation model result. Notes: ^*^ *p* < 0.05, ^**^ *p* < 0.01

The results of regression analyses are listed in Table 5. After controlling for age, stressors in university life were negatively associated with self-efficacy (*β* = -0.24, *t* = -5.51, *p* < 0.01) and positively associated with perceived stress (*β* = 0.38, *t* = 9.68, *p* < 0.01) and anxiety symptoms (*β* = 0.23, *t* = 5.83, *p* < 0.01). The association between self-efficacy and perceived stress was also significant (*β* = -0.24, *t* = -6.13, *p* < 0.01), but not in the association between self-efficacy and anxiety symptoms. Perceived stress had a positive and the strongest association with anxiety symptoms (*β* = 0.51, *t* = 12.85, *p* < 0.01), because the standardized regression coefficient was the largest in the model. Figure 2 represents the model plot after the testing.

**Table 5:** Regression analyses of relationships between variables in the mediation model

Dependent variable	Independent variable	*β*	*t*	LLCI	ULCI	*R*^*2*^	*F*
GSES	Age	0.06	1.50	-0.04	0.28	0.06	16.51^**^
	Stressors in university life	-0.24	-5.51^**^	-0.69	-0.33		
PSS-10	Age	-0.09	-2.24^*^	-0.26	-0.02	0.26	59.16^**^
	Stressors in university life	0.38	9.68^**^	0.57	0.85		
	GSES	-0.24	-6.13^**^	-0.28	-0.14		
GAD-7	Age	-0.04	-1.01	-0.13	0.04	0.40	84.04^**^
	Stressors in university life	0.23	5.83^**^	0.21	0.43		
	GSES	0.06	1.61	-0.01	0.09		
	PSS-10	0.51	12.85^**^	0.33	0.45		

*β* Standardized coefficient, *LLCI* Lower level of the confidence interval, *ULCI* Upper level of the confidence interval

^*^p < 0.05

^**^p < 0.01

The direct, indirect and total effects in the sequential mediation model are shown in Table 6. In the proposed model, stressors in university life impacted anxiety symptoms through four possible routes. The direct effect of stressors in university life on anxiety symptoms (route 1) was 0.32 with 95% bias-corrected CIs [0.21, 0.43] above 0, which was an indication of significance. The mediating effect of self-efficacy (route 2) was not significant, because the 95% bias-corrected CIs [-0.05, 0.01] included 0. Thus, self-efficacy did not play a mediating role in the association between stressors in university life and anxiety symptoms. The mediating effect of perceived stress (route 3) was 0.28 with 95% bias-corrected CIs [0.20, 0.36] excluding 0, supporting the positive mediating effect of perceived stress in the relationship between stressors in university life and anxiety symptoms. Similarly, the sequential mediating effect of self-efficacy and perceived stress (route 4) was 0.04 with 95% bias-corrected CIs [0.02, 0.07] excluding 0, representing the sequential mediating effect of self-efficacy and perceived stress in the relationship between stressors in university life and anxiety symptoms.

**Table 6:** Direct, indirect and total effects in the sequential mediation model

Effect	Route	Coefficient	*SE*	LLCI	ULCI	Percent	
						In mediation effect	In total effect
Direct effect	(1) Stressors in university life → anxiety symptoms	0.32	0.05	0.21	0.43		50.00%
Simple mediation	(2) Stressors in university life → GSES → anxiety symptoms	0	0.01	-0.05	0.01	0	0
Simple mediation	(3) Stressors in university life → PSS-10 → anxiety symptoms	0.28	0.04	0.20	0.36	87.50%	43.75%
Sequential mediation	(4) Stressors in university life → GSES → PSS-10 → anxiety symptoms	0.04	0.01	0.02	0.07	12.50%	6.25%
Total mediation effect	(2) + (3) + (4)	0.32	0.04	0.22	0.38	100%	50.00%
Total effect	(1) + (2) + (3) + (4)	0.64					100%

*SE* Standard error, *LLCI* Lower level of the confidence interval, *ULCI* Upper level of the confidence interval

## Discussion

Previous studies mainly focused on the cross-cultural adaptation of international students, but less on the stressors and related stress responses. In our study sample, the mean scores of PSS-10 was 16.53, which is lower than the local students in Turkey (18.03) [41], Saudi Arabia (20.10) [42] and China (21.13) [43]. In addition, 28.71% of the international students had anxiety symptoms in the present study, which is also lower than the domestic Chinese students (46.85%) [43] and Libyan students (64.50%) [44]. A possible reason may be that the participants in our study all come from a medical university, and they may already have certain amount of knowledge on mental health. It is also possible that the measures taken by their university to manage the stress have been effective.

Our study showed that financial pressure and language barrier were the most serious stressors in university life among international students, which were different from the findings demonstrating that academic difficulties were the primary sources of stress in university students. As pointed out by Grable and Joo, the students who face financial crisis tend to be more likely to drop out of the university or achieve lower grades than others [45], which may cause serious stress to the students. The importance of financial pressure to international students in our study was in line with the previous studies on international students which indicated that the financial pressure was a particular concern and at higher risk for problem of mental health [46, 47]. Language insufficiency has also been found to be a critical stressor that international students encounter in other studies, because language proficiency was essential in international students’ sociocultural adjustment [48, 49]. In this situation, the students may face concomitant problems such as lack of confidence and low self-efficacy, again causing higher level of stress. This finding consisted with the results in previous studies which proved language deficit was a significant source of stress among international students [50, 51]. Furthermore, in our study, stressors in university life were found positively associated with anxiety symptoms of international students (*β* = 0.23, *t* = 5.83, *p* < 0.01), which supported H1 and was consistent with other studies [52]. Since the students are exposed to various stressors in university life to different extent and it may not be possible to remove the stressors from their roots, understanding the internal mechanism becomes very important in order to reduce the adverse effect of stressors in university life and maintain the mental health of students.

In the present study, stressors in university life were negatively associated with self-efficacy (*β* = -0.24, *t* = -5.51, *p* < 0.01) and positively associated with perceived stress (*β* = 0.38, *t* = 9.68, *p* < 0.01), which supported H2 and H5. Self-efficacy was negatively associated with perceived stress (*β* = -0.24, *t* = -6.13, *p* < 0.01), and perceived stress was positively associated with anxiety symptoms (*β* = 0.51, *t* = 12.85, *p* < 0.01), which supported H8 and H6. Unexpectedly, sequential mediation model testing didn’t show a direct effect of self-efficacy on anxiety symptoms nor an indirect effect of self-efficacy in the association between stressors in university life and anxiety symptoms. Therefore, H3 and H4 were not supported, which indicated that the association of self-efficacy with anxiety symptoms was not direct, similar with the findings from a study on medical college students in Philippines [53]. Instead, self-efficacy played a sequential mediating role with perceived stress in the association between stressors in university life and anxiety symptoms, which supported H9 and indicated self-efficacy’s direct relationship with perceived stress rather than anxiety symptoms. Although the sequential mediation effect accounted only for 12.5% of the total mediation effect, it still implied that the impact of self-efficacy on anxiety symptoms was generated through perceived stress. This result supported the transactional model of stress. It also indicated that self-efficacy was an effective protective factor against stress. Individuals who have lower levers of self-efficacy do not have enough confidence and the ability to cope with the external and internal environment. They will perceive more severe stressors and stress, and are more prone to show anxiety symptoms. Self-efficacy improvement interventions in previous researches have shown that the methods were effective in empowering participants to cope with stress [22, 24, 54–56].

Another finding of our study was the partial mediation effect of perceived stress in the association of stressors in university life and anxiety symptoms among international students, which supported H7. Perceived stress alone accounted for 87.50% of the total mediation effect and 43.75% of the total effect. Its strong effect indicated its important role in facilitating translation of stressors in university life into anxiety symptoms, and this is in line with other studies that assumed appraisals were important determinants of adjustment to stressful encounters [57]. Previous empirical researches have shown similar findings of the mediation effect of perceived stress [25–27]. Combined with our findings of self-efficacy as a protective factor against stress, interventions can be considered using self-efficacy training to alleviate perceived stress and promote the positive appraisal on stressors in university life to reduce anxiety symptoms. There already have been some researches of stress management among university students using cognitive behavioral therapy which have achieved a significant reduction in perceived stress and anxiety symptoms after the intervention, with the enhancement of self-efficacy as well [17].

### Limitations

Our study has several limitations. Given the cross-sectional design, it’s unable to make any assertions regarding causation. A further experimental design of study in the future should be employed to determine causal relationships. Another limitation is that there may have been response biases in the self-report of the individuals completing the measures. Finally, as most of the participants were from Asia, the results in this study may not apply equally well to the students in other part of the world. Future research could expand the diversity of the university types to better capture the students from other part of the world.

Despite of the limitations, this study has discovered the sequential mediating role of self-efficacy and perceived stress in the association between stressors in university life and anxiety symptoms, and provided a new perspective on how to maintain mental health for international students. The sequential mediators provides a deeper insight into the underlying mechanism of stressors in university life towards anxiety symptoms among international students. At the same time, this study has broadened the application scope of self-efficacy in the field of stress research, and is also an empirical contribution to the theory of transactional model of stress using in the population of international students. In addition, our study shows that identification and evaluation of stressors in university life are important, and financial pressure and language barrier should be given more attention for international students. This would be valuable in ensuring implementation of stress reduction programs to effectively support students. Given that few studies on university life stressors exist in the literature of international students, our study is very important because it has filled in the gap. As for practical implications, our study findings may apply to all the international students who have poor self-efficacy or perceive higher levels of stress or are struggling with anxiety. Therefore, counselling focusing on financial pressure and language barrier, as well as introduction of specific interventions into university campus for international students should be encouraged, and the university educators should utilize self-efficacy improvement and stress reduction measures in the training programs to support students.

## Conclusions

Our study has identified the financial pressure and language barrier as the most important university life stressors for international students. The findings have also confirmed the direct positive association between stressors in university life and anxiety symptoms, as well as the positive association between perceived stress and anxiety symptoms, and revealed the sequential mediating role of self-efficacy and perceived stress in the association between stressors in university life and anxiety symptoms. Results of our study indicate that in order to maintain the mental health of international students, counselling concerning finance and language and interventions with self-efficacy improvement and stress reduction should be involved in the training programs within the university campus.

## Data Availability

The datasets generated and/or analyzed during the current study are not publicly available due to the protection of participants' privacy but are available from the corresponding author on reasonable request.

## Abbreviations

GAD-7: Generalized Anxiety Disorder 7-item
GSES: General Self-efficacy Scale
PSS-10: 10-Item version of Perceived Stress Scale
